# Transcriptome analyses and differential gene expression in a non-model fish species with alternative mating tactics

**DOI:** 10.1186/1471-2164-15-167

**Published:** 2014-02-28

**Authors:** Celia Schunter, Steven V Vollmer, Enrique Macpherson, Marta Pascual

**Affiliations:** 1 Centre d’Estudis Avançats de Blanes (CEAB-CSIC), Car. Acc. Cala St. Francesc 14 Blanes 17300 Girona, Spain; 2 Dept. Genètica, Univ. Barcelona, Av. Diagonal 643, 08028 Barcelona Spain; 3 Marine Science Center, Northeastern University, 430 Nahant Road, Nahant, MA 01908, USA

**Keywords:** RNAseq, Differential expression, Alternative mating tactics, Social dominance, Phenotypic plasticity, *Tripterygion delaisi*

## Abstract

**Background:**

Social dominance is important for the reproductive success of males in many species. In the black-faced blenny (*Tripterygion delaisi*) during the reproductive season, some males change color and invest in nest making and defending a territory, whereas others do not change color and ‘sneak’ reproductions when females lay their eggs. Using RNAseq, we profiled differential gene expression between the brains of territorial males, sneaker males, and females to study the molecular signatures of male dimorphism.

**Results:**

We found that more genes were differentially expressed between the two male phenotypes than between males and females, suggesting that during the reproductive period phenotypic plasticity is a more important factor in differential gene expression than sexual dimorphism. The territorial male overexpresses genes related to synaptic plasticity and the sneaker male overexpresses genes involved in differentiation and development.

**Conclusions:**

Previously suggested candidate genes for social dominance in the context of alternative mating strategies seem to be predominantly species-specific. We present a list of novel genes which are differentially expressed in *Tripterygion delaisi*. This is the first genome-wide study for a molecular non-model species in the context of alternative mating strategies and provides essential information for further studies investigating the molecular basis of social dominance.

## Background

Polygamous mating systems are often defined by social dominance where territorial individuals top the social hierarchy. Alternative mating strategies in fish species are commonly associated with a dominance hierarchy including dominant or territorial males and secondary males or so-called sneaker males [1]. Being the dominant individual comes at a cost, as the territorial male has to invest in defending the territory, attract the female and guard the nest [2]. In some fish species those dominant individuals even do not feed during the reproductive period of several months [3]. The sneaking individual on the other hand can obtain reproductive success by sneaking into nests or mimicking female behavior and phenotype [4].

The term ‘social dominance’ suggests that dominance depends on the social setting. The territorial male is often the largest male present in many fish species but there are exceptions [5,6]. This is especially true for fish species where the change from sneaker to territorial male is not fixed for life, but temporary and therefore plastic. Here, the switch to becoming territorial could depend on the presence of plausible nest sites, the number of mature females or even the presence or absence of other males [4]. For instance, in the goby *Gobius niger*[7] and the blenny *Tripterygion delaisi*[8,9] a sneaker male can switch into a territorial male after the experimental removal of the previous territorial male.

Phenotypic plasticity, the ability of a genotype to respond to external conditions by changing its phenotype, has received considerable attention in evolutionary ecology [10]. The assimilation of an initially plastic response to an altered environment and therefore the maintenance of the phenotype has long been understood as a fundamental component in selection and evolution [11,12]. More recently, short-term and non-adaptive phenotypic changes have been the focus of several studies [13,14]. In many species reproduction is a temporal or a short-term event, taking place for example only once a year in the reproductive period. This means that the alterations occurring during this period, being behavioral and/or phenotypic, are plastic and mostly reversible changes [15]. In male alternative mating tactics, the different tactics are linked with differences in behavior often leading to phenotypic dimorphism with secondary sexual traits. Social interactions, in these cases, have been shown to trigger the behavioral and phenotypic change [16].

Social influences and behavioral changes lead to alterations in gene expression in the brain [17-19]. More specifically, social stimuli can lead to short term deviation from the baseline of gene expression in the brain [17]. While it is unclear how these changes are transmitted to the other organs, clearly the brain plays a vital role [20]. The neural basis of social status has been studied in the brain in Atlantic salmon (*Salmo salar*) where the plasticity in the development of alternative male phenotypes has been investigated [21,22]. Aubin-Horth and coauthors [21] discovered that 15% of the analyzed genes were differentially expressed between brains of male morphs, but the investigations into alternative mating tactics in the Atlantic salmon were carried out on precocious males and lack the analysis of the large mature male. Alternative male mating strategies are also studied in cichlid fish species, due to their extreme diversity and the facility to be handled and kept in the laboratory (see [4] and references cited therein), whereas the non-dominant male is not reproductively active [19]. Gene expression patterns related to phenotypic plasticity in different mating strategies have been analyzed either by targeting single genes or via microarray analyses [14,19,21]. To our knowledge up to now no attempt to characterize phenotypic plasticity in wild male phenotypes from the same population, both reproductively active, has been carried out using a genome-wide approach.

Our study species *Tripterygion delaisi* (Tripterygiidae), also called the black-faced blenny, is a common small rocky shore fish from the Mediterranean Sea and the east Atlantic coast [23,24]. The black-faced blennies live camouflaged with the rock or algae they inhabit for most of the year. The sneaker males as well as the females exhibit the same camouflaged phenotype throughout the whole year, but in spring throughout the three months reproductive period, some males change their phenotype to a black head and a bright yellow coloring across the rest of the body. These males start protecting a small territory, which is referred to as their nest, against predators and other secondary males [8]. This coloration and behavior is transitory and only observed during the reproductive period. If a territorial male is removed from its nest, in 20% of the cases, a sneaker male takes over and changes its coloration as well as its behavior and becomes territorial [9]. This shows that territoriality is a plastic trait. After being courted by the territorial male, the female lays the eggs directly into the nest and leaves. The territorial male fertilizes the eggs by releasing the sperm directly on them and is left to protect the eggs until the larvae hatch. The sneaker male can dart by and ejaculate its sperm over the nest from a distance [8]. Thus, in *T. delaisi* alternative mating tactics can be observed for two types of reproductively active males with different phenotypes.

In non-model species, it is often difficult to study molecular differentiation of plastic traits especially in absence of a reference genome [25]. Here we used RNAseq to detect differentially expressed genes in the brain between males with alternative mating strategies in *Tripterygion delaisi* as well as between males and females. By sequencing and generating a *de novo* transcriptome assembly it is possible to look at a huge variety of expressed genes and demonstrate key genes which are expressed at a particular moment [26]. By using RNAseq in this study we generate a genome-wide catalogue of genes expressed during the reproductive period of *T. delaisi* and analyze expression profile and differential expression to identify differences across the brain transcriptome of territorial males, sneaker males and females.

## Results and discussion

### De novo transcriptome assembly and annotation

In the absence of a reference genome for *Tripterygion delaisi* in this study we *de novo* assembled a transcriptome to use as a reference for read mapping and brain gene expression profiling. The *de novo* assembly of the reference brain transcriptome was performed with 50,360,654 trimmed reads (Phred score 35) of eight pooled individuals of normalized cDNA libraries and 38,056,381 trimmed reads of three separately sequenced not-normalized samples at 109 bp (Additional file 1: Table S1, Additional file 2: Figure S1). Normalizing libraries allowed for the detection of genes also expressed at low levels and therefore a more complete reference transcriptome. With the Trinity *de novo* assembler 328,565 contigs were produced, including different isoforms per contig, after removing contamination (see Methods section). The N50 of the reference transcriptome is 298 bp long, the N25 234 bp and the N75 is of 464 bp length. The longest contig has a length of 15,547 bp. On average 76% of the reads of the individual samples were mapped back onto the reference assembly. This is the first reference transcriptome for a fish belonging to the Perciforms Suborder Blennioidei (which includes more than 850 species and 151 genera [27]) and therefore provides a valuable resource and a first step towards a comprehensive understanding of genome-wide gene expression.

The *de novo* assembly includes differentially spliced isoforms [28]. Most contigs in the reference assembly had one isoform, but many contigs combined differentially spliced isoforms, with the number of isoforms increasing with contig length (Additional file 3: Figure S2). However, given the lack of a reference genome, we cannot discard that some isoforms might result from paralogous genes or misassemblies. The few published genome-wide expression studies on non-model species primarily focus on differential expression at the gene level and *de novo* assemblies do not include alternatively spliced transcripts e.g. [29]. However, alternative splicing has fundamental effects in the development and maintenance in eukaryotes with 92-94% of human genes undergoing alternative splicing [30,31]. The products of alternatively spliced transcripts are shown to be responsible for a number of diseases by changing the biological function with differently spliced isoforms [32]. Hence, it is likely that even social behavior or phenotypic expression patterns could be influenced or even dominated by alternative splicing.

**Figure 1:**
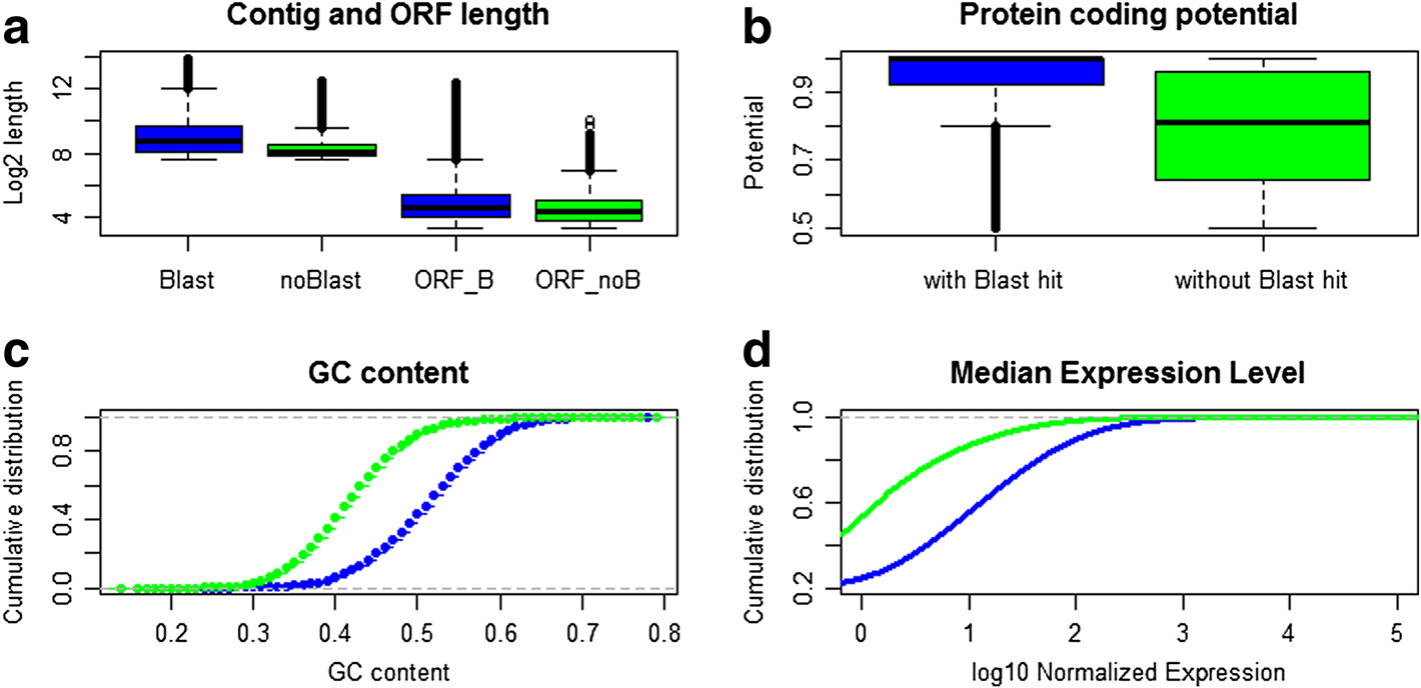
**Comparison between the contigs with BLAST-hits and the set of contigs without BLAST-hits. a)** Overall length and ORF length, **b)** Protein coding potential determined by CPAT, **c)** GC content per contigs, **d)** Overall normalized expression.

Similarity with known proteins (<E-values 1×10^-10^) was detected for 71,513 contigs which represent 21.4% of the *de novo* reference transcriptome assembly. Overall 67,165 contigs had BLAST results between 1×10^-11^ to 1×10^-180^ and 4348 contigs had an E-value of 0. Hence, many of the contig sequences could not be matched to a protein and therefore to a functional description in the protein databases. There can be different reasons for the lack of homology with known proteins. These contigs could be orphan proteins, non-coding RNA or sequences from UTR protein regions, although, we cannot discard the presence of partially or misassembled transcripts. Furthermore, lack of sequence conservation across species combined with absence of genomic information for *T. delaisi* could prevent the annotation of contigs. When blasting (BLASTn) the longer contigs (>750 bp, 40612 contigs) against the Refseq database of *Oreochromis niloticus* (closest relative to *T. delaisi* in GenBank) we receive significant hits (e-value 10^-5^) for over 89 percent. For further descriptive analysis we divided our transcriptome contig set into two subsets: contigs with protein homology (with BLAST) and contigs without protein homology (without BLAST). The ‘with BLAST’ set had slightly larger contig sizes than the ‘without BLAST’ set (Figure 1a). Similarly, median open reading frame (ORF) sizes, measured in nucleotide bases, differed slightly between sets, with larger ORFs in the ‘with BLAST’ than in the without BLAST set (‘with BLAST’: 114.9, ‘without BLAST’: 80.31). Protein coding potential above 0.5 was estimated with CPAT [33] and the without BLAST set has lower protein coding potential (Figure 1b). Higher GC content as well as higher expression levels were encountered for the ‘without BLAST’ set (Figure 1c,d). The described characteristics of the ‘without BLAST’ set in *Tripterygion delaisi* have also been reported for the wasp [34] which is also a species without a reference genome. In general, more genomic resources for *T. delaisi* or closely related species and more information on protein functions would increase the quality of the transcriptome assembly.

**Figure 2:**
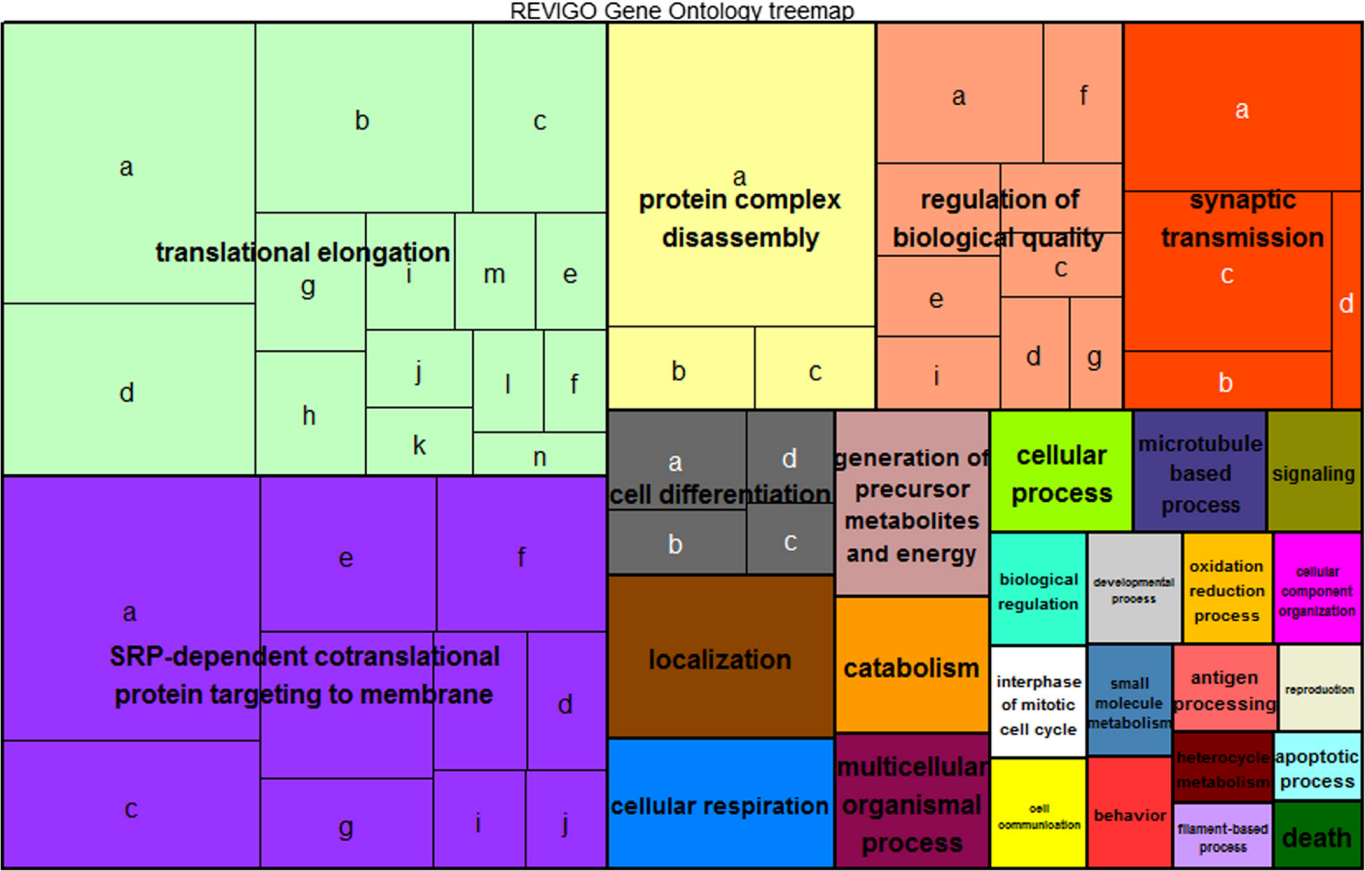
**Gene Ontology treemap for the 1% highly expressed genes regardless of phenotype.** The box size correlates to the –log10 p-value of the GO-term enrichment. Boxes with the same color can be grouped together and correspond to the same upper-hierarchy GO-term which is found in the middle of each box. Description of letters can be found in Additional file 1: Table S2.

The most expressed genes from the transcriptome (first 1% of mean normalized expression) regardless of phenotype were annotated for biological function in BLAST2GO [35] and enrichment analysis resulted in upregulated expression of 134 slimmed GO-terms in biological function. The majority of these terms (about 1/3) is involved in processes of translation and transport such as translation elongation and SRP - dependent cotranslational protein targeting to membrane (Figure 2, Additional file 1: Table S2). Interestingly, one of the twenty-eight enriched upper-hierarchy GO-terms is behavior. When looking at the first ten genes with the highest overall expression count across the whole genome, we identified a gene called Ependymin (*epd1*) (see Additional file 1: Table S3). This gene is predominantly expressed in the cerebrospinal fluid of teleost fish [36] and with our results we confirm that this gene is highly important in the brain of *Tripterygion delaisi*. Ependymin is associated with neuroplasticity and regeneration and might indicate a stress response [37]. A limiting factor in the study of gene expression of wild-caught animals is the introduction of stress which can lead to behavioral alterations.

### Differential expression between phenotypes

**Figure 3:**
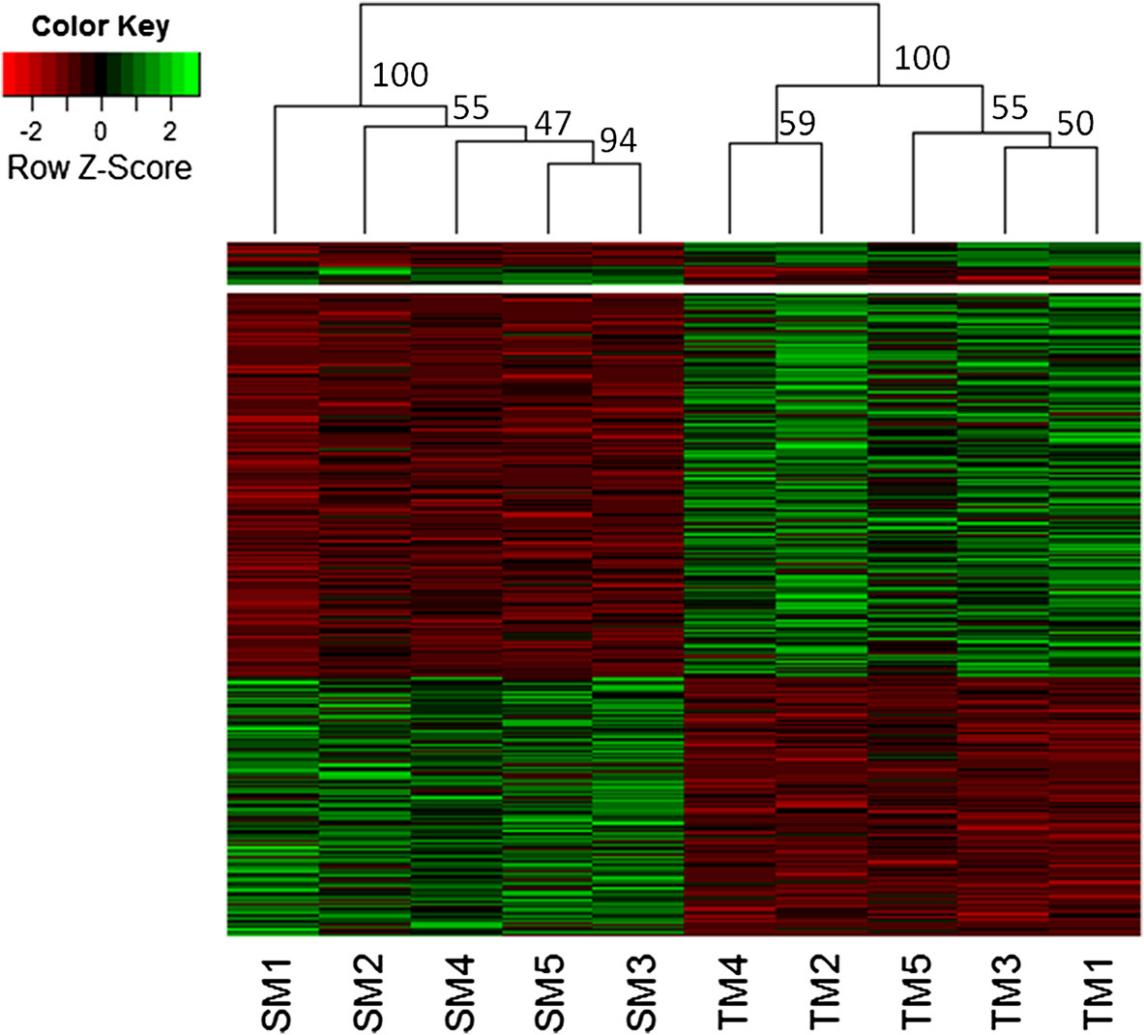
**Heatmap comparing significant differentially expressed contigs, either annotated or not annotated, between five sneaker males and five territorial males.** Intensity of color indicates expression levels. Similarity in expression patterns between genes is represented by kmeans clustering separating highly expressed genes above the white line and less expressed genes below. Similarity between individuals with hierarchical clustering can be seen above the heatmap with bootstraps.

The RNAseq methodology based on 15 individuals allowed a genome wide analysis of the expression patterns that distinguish the three phenotypes of *Tripterygion delaisi* using five territorial males, five sneaker males and five females. On average 13 million quality trimmed reads were used for each of the 15 individuals (Additional file 1: Table S1). Three pairwise comparisons were performed by using a Bayesian approach to accurately estimate isoform expression (for details see Materials and Methods). Individual variation was accounted for by only accepting significant differential expression if standard deviation was smaller than the mean expression value within each phenotype. The final set of differentially expressed contigs between phenotypes is represented by hierarchical clustering of the expression patterns (Figure 3, Additional file 4: Figure S3 & Additional file 5: Figure S4). The territorial male differentially upregulated more genes than the other two phenotypes when all three comparisons were considered (Table 1), possibly indicating that expression of an elevated number of genes are necessary for the maintenance of this phenotype. The comparison between the territorial male and the sneaker male resulted in the highest number of differentially expressed contigs with 600 significant contigs after FDR correction and adjustment for individual variation (Table 1, for a list of all genes see Additional file 1: Tables S4, S5 and S6). For these differentially expressed contigs between the two male phenotypes, territorial males and sneaker males are separated into two clusters illustrated by the hierarchical distance tree (Figure 3), representing the genes involved in phenotypic plasticity of different social statuses. In general, more differentially expressed genes were found for the comparison between the two male phenotypes than between males and females. This suggests that phenotypic plasticity, rather than sexual dimorphism, causes greater differences in gene expression patterns between phenotypes during the reproductive period of *T. delaisi.*

**Table 1:** Number of significantly expressed contigs and percentage of annotated genes between phenotypes and for a given phenotype with all comparisons combined

	**Total**	**Annotated**
**TM > SM**	360	21.90%
**SM > TM**	240	54.20%
**TM > F**	144	43.10%
**F > TM**	104	28.80%
**SM > F**	162	57.40%
**F > SM**	177	26.00%
	**Over-expressed**	**Annotated**
**TM**	504	28.00%
**SM**	402	55.50%
**F**	281	27.00%

FDR adjusted significance value of 0.05. > indicates elevated expression in the phenotype on the left. TM = territorial male, SM = sneaker male, F = female.

**Figure 4:**
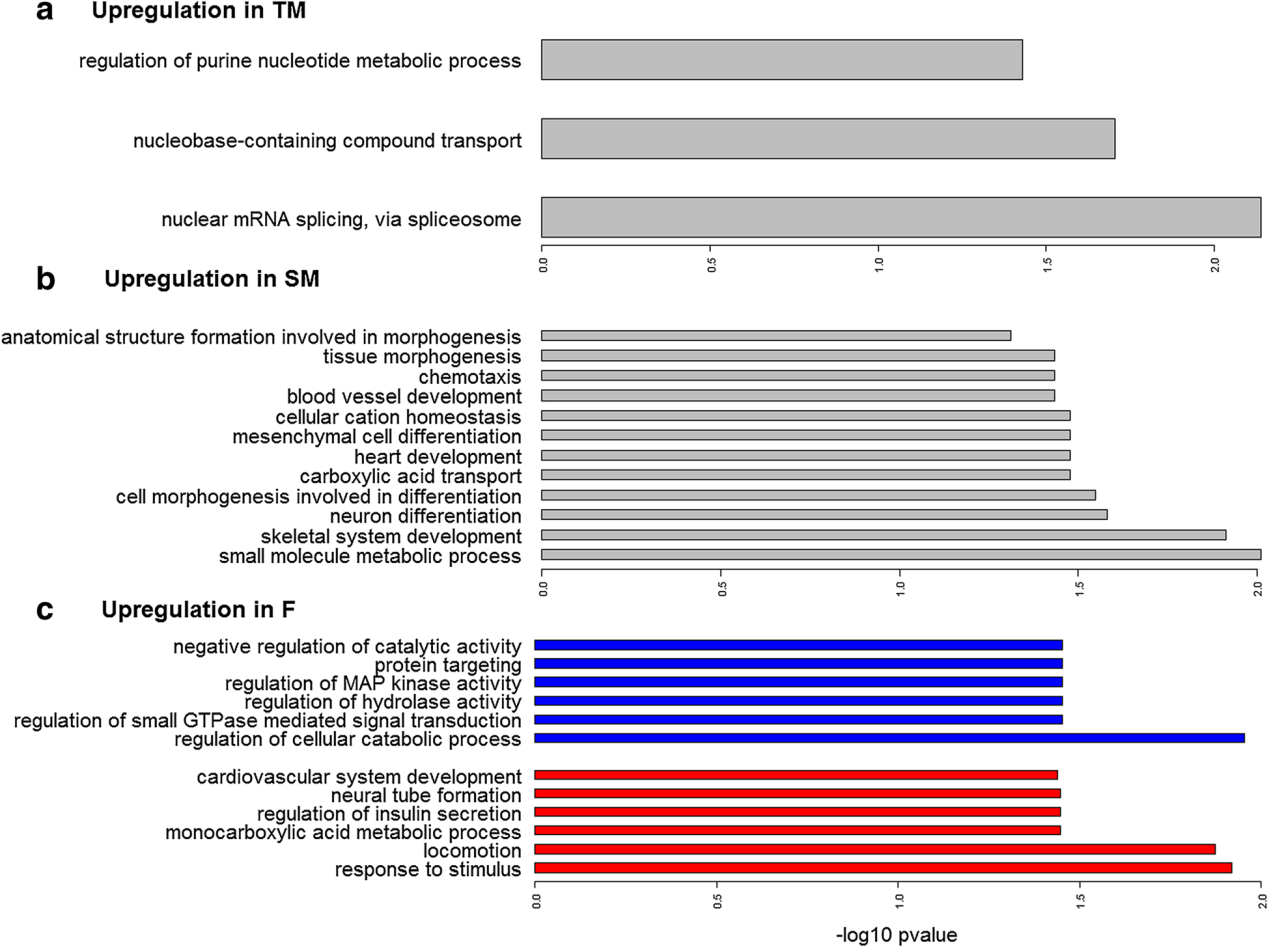
**Barcharts represent the enriched biological processes associated with the upregulated genes in SM and TM (a, b), whereas no enrichment was found with upregulation against females.** Upregulation in females **(c)** against the territorial male (red) and sneaker male (blue).

Phenotypic plasticity has received considerable attention in evolutionary ecology, whereas focus has been laid on long term adaptive phenotypic changes [10,38]. Recent studies, based on model species, which test the more rapid time scale of response to environment such as temperature, light, and presence of pathogens or pheromones [38-40] have demonstrated an important role for protein phosphorylation. Protein kinases, such as those involved in the mitogen-activated protein kinase (MAPK) signaling pathway, mediate phosphorylation changes in other proteins and have been implicated in the control of synaptic plasticity in the adult brain [41]. Genes from the map kinases pathway which are associated with social phenotypic plasticity were identified as differentially expressed in *T. delaisi. Madd,* for instance, was upregulated in the territorial male in two different isoforms against the sneaker male whereas *mapkapk3* was upregulated for the sneaker male against the territorial male (Additional file 1: Table S4). Furthermore, we find regulation of MAP kinase activity to be enriched in the female (Figure 4). This enrichment may be due to several isoforms of trib2, a gene that interacts and regulates activation of MAP kinases, being overexpressed towards the sneaker male. Thus, these genes might be worthwhile analyzing more profoundly in future studies.

Annotation, meaning contigs with information on functional protein characteristics, was highest in sneaker males (55.5%, Table 1). For females and territorial males only about one third of the upregulated contigs could be annotated to known proteins (27% and 28% respectively). The enriched GO-terms for the sneaker male are predominantly involved in differentiation and development (Figure 4). This could suggest that the elevated annotation success for the upregulated genes in sneaker males might be related to the presence of more functional descriptions of these developmental genes in the databases. On the other hand, over 70% of differentially expressed genes are left unannotated to a known protein for the territorial male and the female and especially low annotation success was found for the upregulated genes in territorial males against sneaker males (Table 1). As stated above, general reasons for non-annotation of contigs could be lack of sequence conservation, non-coding RNA and orphan genes. However, here by comparing the annotation rate of the phenotypes directly, the results might indicate that the genes involved in social phenotypic plasticity are poorly studied and therefore lack information of the protein function in the databases.

### Candidate genes in the context of social behavior

Several previous studies have proposed candidate genes for social behavior and dominance in other fish species with alternative mating tactics [41], for details please see Material and Methods]. All genes were present in all *Tripterygion delaisi* individuals and the expression levels of these selected genes were compared between phenotypes (Additional file 1: Table S7). The only candidate gene that showed significant differences in expression after correction for individual variation in *T. delaisi* was the Somatostatin receptor type 1 (*sstr1*) between territorial males and females. Somatostatin is a neuropeptide also known as a growth hormone-inhibiting hormone and therefore commonly studied in the context of growth. In the African cichlid (*Astatotilapia burtoni*) somatostatin and somatostatin receptors have been shown to play a role in social behavior [42-44]. Somatostatin prepropeptide and somatostatin receptor type 3 (*sstr3*) were elevated in the dominant cichlid males in comparison to the subordinate males. For *T. delaisi*, differential expression was found for *sstr1,* which was not measured in cichlids, with significant differential expression between territorial males and females (and intermediate expression levels for the sneaker male). It is probable that elevation in *sstr1* levels for *T. delaisi* males and especially territorial males is correlated with aggression. For cichlids, somatostatin has been shown to have a significant effect on aggressive behavior and aggression levels were correlated with *sstr* (2 and 3) expression in the gonads [43]. This is the first evidence for the role of *sstr1* in association with different morph types demonstrating that the effects of somatostatin on the regulation of growth and behavior are complex [43].

No other previously suggested candidate genes for social dominance were differentially expressed at a significant level in *Tripterygion delaisi* with the exception of the brain aromatase enzyme (*cyp19a1*), which catalyzes the formation of aromatic estrogen. For this gene multiple isoforms were differentially expressed but only before adjustment for individual variation (Additional file 1: Table S7). *Cyp19a1* was found at higher levels in territorial males in comparison to sneaker males in other fish species [e.g. [15]. Aromatase is duplicated in fish and one form is expressed in the ovaries and the other one is expressed in the brain. Brain aromatase activity was found to be lower in castrated males than in non-castrated males of *Salmo salar*[45] and lower in individuals with developing gonads in comparison to individuals with fully developed gonads in Atlantic croaker (*Micropogonias undulates*; [46]). In peacock blennies aromatase activity was also suppressed in sneaker males and elevated in nesting males [1]. Although this shows that brain aromatase clearly is an important enzyme involved in the regulation of social status between males in several fish species, individual variation of the expression of this gene was high in *T. delaisi* thus we don’t consider this gene differentially expressed (Additional file 1: Table S7). The five sneaker males all expressed the *cyp19a1* isoforms at an equally low level, whereas two of the five territorial males show very high levels resulting in differential expression between the male types due to these outliers. This result could be attributed to slight differences in the reproductive phase of the territorial males (e.g. time since/until sperm ejaculation, female presence). Individual gene expression variation has previously been pointed out to be an important factor for phenotypic plasticity [14] and outlier expression might bias the outcome [29]. Pairing careful behavioral studies with genome wide molecular analyses could therefore strengthen the conclusion, but this especially emphasizes the need of non-pooled biological replicates even in genome wide studies.

A commonly measured neuropeptide in relation to aggression, arginine vasotocin (*avt*), is differentially expressed between males in several fish species, but not in *Tripterygion delaisi* (Additional file 1: Table S7). In Atlantic salmon (*Salmon salar*) vasotocin is one of the key genes that is differentially expressed in the brain with down-regulation for the precocious male [22] as in the peacock blenny (*Salaria pavo*) where *avt* levels were detected to be higher in territorial nesting male in the forebrain [47]. In the African cichlid (*Astatotilapia burtoni*) elevated expression of *avt* was found in the dominant male in the posterior preoptic area and in the anterior preoptic area *avt* mRNA levels were higher in the non-dominant male [48]. Such regional expression differences were also observed in three-spined stickleback (*Gasterosteus aculeatus*) in relation to territoriality [49]. The fact that whole brain expression was measured in *T. delaisi* might mask actual expression differences between different brain regions. Nonetheless, Santangelo & Bass point out that there might be a species and context dependency of *avt* regulation across teleost species as seen for tetrapods [50].

The fact that most of the previously mentioned candidate genes were not differentially expressed in *Tripterygion delaisi* could be due to slight differences in reproductive social system. In African cichlids, the subordinate males have undeveloped testes and need to become territorial to reproduce [19], which is distinct to the sneaker male in *T. delaisi.* Testes in *T. delaisi* sneaker males are significantly different in weight (Unpaired t-test: t = 5.089, p = 0.0002), on average they weighed 1.78 times more than those of the territorial males. This shows that the sneaker male has proportionally greater testes and is reproductively active. In Atlantic Salmon precocious males and adult males show differential expression in some of the candidate genes but the two male types reproduce at different ages and the adult males do not settle down and defend a nest [21]. This suggests that the regulation of social and reproductive behaviors is complex, and consideration of expression patterns for candidate genes should take species-and context-specific differences into account.

### Novel genes in the context of social behavior

**Figure 5:**
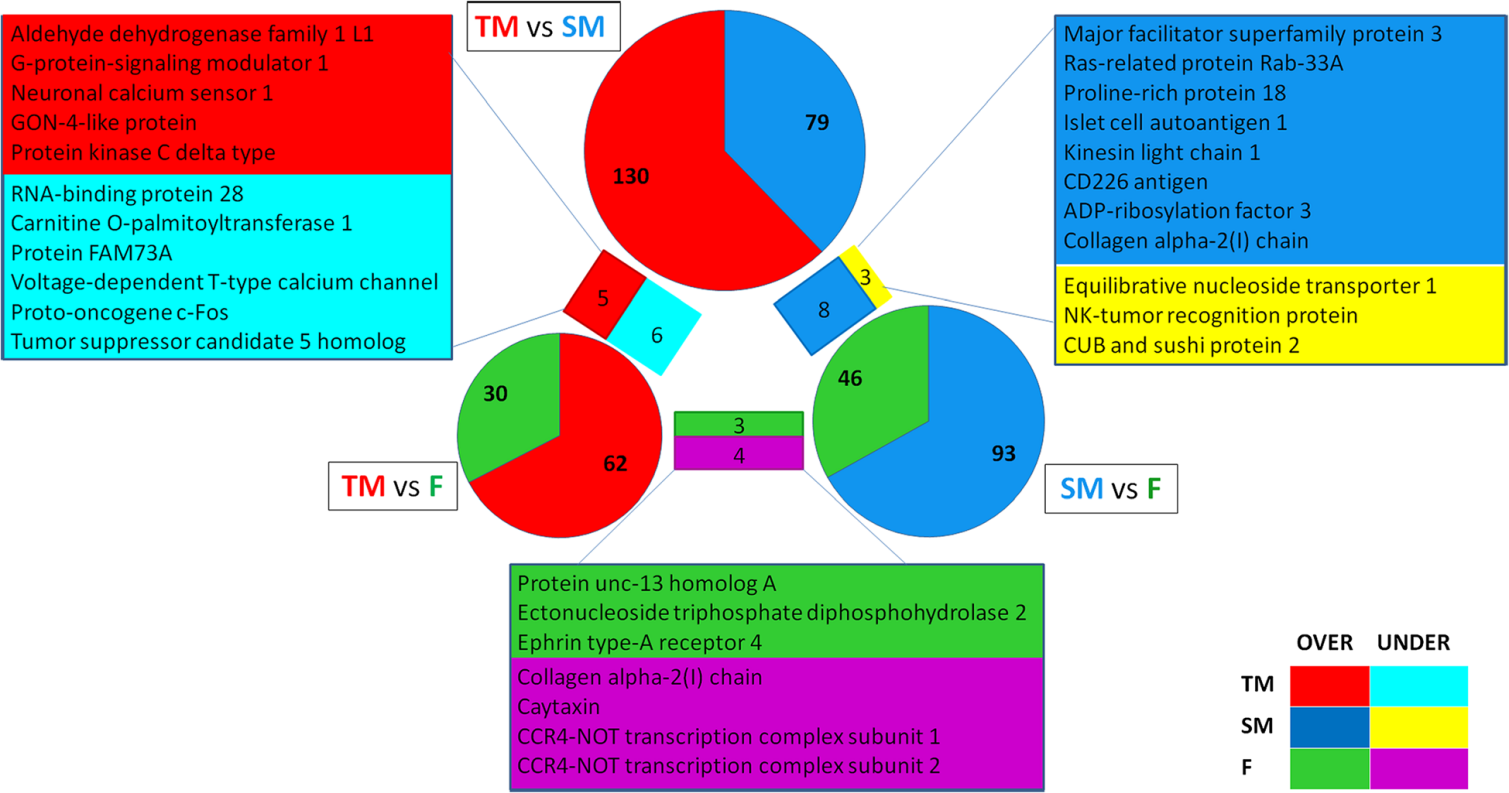
**Venn diagram of the three comparisons: territorial male versus sneaker male (TM vs SM), territorial male versus female (TM vs F) and sneaker male versus female (SM vs F).** The circle size is scaled to the number of differentially expressed genes for each comparison. Bars between piecharts and the corresponding gene lists identify contigs which are significantly expressed in the two adjacent comparisons and therefore representative of one phenotype. These genes are presented in the squares boxes and the colors identify over or under expressed in the different phenotypes. In red is the elevated expression in territorial male, in blue elevated expression in sneaker male and in green elevated expression in female. Significant expression at lower levels in territorial males is shown in turquoise, pink are the genes expressed at lower levels in females and yellow are expressed at lower levels in sneaker males.

By RNAseq analysis we uncovered a large set of differentially expressed novel genes with protein annotation during the reproductive period in *Tripterygion delaisi*. Key genes with a functional description in the databases could be detected for each of the phenotypes (Figure 5). When comparing the females with the two types of males three (annotated) genes are expressed at higher levels in females and four in the two types of males. Two subunits of the CCR4-NOT transcription complex, which function as a general transcription regulation, are more expressed in both male phenotypes in comparison to the females. The function of the CCR4-NOT complex is involved in all aspects of mRNA biogenesis from the transcription of RNA to its export [51]. This could be correlated with lower transcriptional activity in females, as less upregulated genes are found in females (Figure 5). *Col1a2*, which encodes for Collagen Type I alpha, is expressed in higher level in both *T. delaisi* males relative to the females. This gene is a well-studied gene in the context of bone development [52]. In the brain tissue, collagen can only be found in the blood vessels of the brain, which either suggests development of blood vessels or a possible transport of collagen to other organs for skeletal development. Both of these biological functions are enriched for the sneaker male (Figure 4).

It is important to not only focus on enrichments based on GO analyses, but to individually look at the genes of interest, as certain patterns could be drawn. The genes defining the sneaker male in *T. delaisi* mostly have functions related to transport. *Rab33a*, *arf3* and *mfsd3* are associated with protein transport, protein trafficking and vesicle transport. *Klc1*, kinesin light chain 1, is responsible for organelle transport along microtubules. For the territorial male some genes related to synaptic plasticity show elevated expression such as *gpsm1*, a G-protein signaling modulator and *ncs1*, a neuronal calcium sensor, both involved in the activation or deactivation of the G-protein cascade. The *gpsm1* gene acts as a sink to regulate availability and stability of Gα in the G-protein pathway [53]. This Gα component mediates signaling from vasoconstrictive hormone, such as vasopressin (homologue for vasotocin in fish) [54], a previously mentioned candidate gene for aggression in fish [55]. The neuronal calcium sensor (*ncs1*), which is also upregulated in the territorial male, is sensitive to cytosolic Ca^2+^ changes and contributes to G-protein-coupled receptor desensitization and increases vesicle release in the presence of calcium. As *ncs1* was found in the dendroids in mice it may allow for locally regulated protein synthesis, which is linked to long-term synaptic plasticity [56]. Also upregulated in the territorial male is the protein kinase Cδ (*prkcd*) which is a recently-detected PKC isoform that plays critical roles in various cellular functions such as the control of growth, differentiation, and apoptosis [57]. In rodents, though, *prkcd* is a gene involved in signal transduction that is correlated with behavior [58]. The territorial male also expressed *Aldh1l1* at elevated levels which encodes for an enzyme from the aldehyde dehydrogenase family. A gene from this family (*aldh9*) is one of the few genes that was associated with dominance of African cichlid males in a microarray study [14]. Although in cichlids this gene was expressed in lower levels for the dominant male against the subordinate male, it might be interesting to study this gene family directly in relation to behavior.

### Importance of alternative splicing in the context of social dominance

The importance of alternatively spliced gene isoforms has long been accepted [31] and with the development of RNAseq a more detailed understanding is possible. Although most studies using RNAseq focus on expression on the gene level, expression of differentially spliced isoforms might vary even though overall gene expression might not. For humans 10% of the protein coding genes reveal population-specific splicing [59]. For *Tripterygion delaisi* nuclear mRNA splicing via the spliceosome is one of the three enriched biological functions differentially expressed for the territorial male, indicating the elevated importance of exon joining and possibly alternative splicing in territorial males (Figure 4). Nonetheless, for the differentially expressed genes in the context of social dominance, most alternatively spliced isoforms showed the same expression pattern (for details see gene tables in Additional file 1: Table S4, S5, and S6). However, five differentially expressed genes with functional annotations in the databases were expressed in an opposing manner in two isoforms between phenotypes. An isoform of *phf20* was more highly expressed in the territorial male and a different isoform in the sneaker male. Two isoforms of the genes *prkar2b* and *c7orf51* showed opposing expression patterns in the comparison between sneaker male and female, and different isoforms for the genes *rap1gap* and *macf1* presented contrasting expression patterns between the territorial male and female. In the case of *rap1gap* evidence shows that differentially spliced isoform transcript variants encode distinct proteins leading to different functions e.g. [60]. These genes would have been overlooked as not differentially expressed if only expression at the gene level was considered. However, due to the complexity and lack of complete understanding for the patterns of alternative splicing, it is still a major challenge to identify differential expression of isoforms and results need to be interpreted with caution. Nonetheless, advances in analysis methods for RNAseq, such as the assembly of clusters of full length alternative spliced isoforms [28] and models accommodating isoform expression estimates uncertainties [61], allow the estimation of differential gene expression for isoforms in our species. Even though more work is needed to fully understand the precision and accuracy as well as the possibilities and limitations of the methodologies used, this approach will allow for gene expression studies in non-model species of evolutionary and ecological importance.

## Conclusions

Phenotypic plasticity was found to be a more important factor in differential gene expression during reproduction than sexual dimorphism as more genes were significantly expressed at different levels in the brain between the two male phenotypes than between males and females. Previously suggested candidate genes for social dominance in the context of alternative mating strategies seem to be mostly species-specific and here we present a list of novel genes which are differentially expressed in *Tripterygion delaisi*. Some genes that were differentially expressed for the territorial male were related to synaptic plasticity possibly indicating the drastic change in behavior and phenotype. The sneaker male expresses genes associated with differentiation and development. This result suggests that although this type of male is reproductively active it is largely expressing genes involved in development. This is the first study looking at transcriptome data and differential expression for a non-model species (*T. delaisi*) in the context of alternative mating strategies and provides essential information for further studies investigating the molecular basis of social dominance. Overall, RNAseq has proven to be a useful tool for the analysis of ecological and evolutionary questions for non-model species.

## Methods

### Sample collection

Territorial males, sneaker males and females of *Tripterygion delaisi* were collected in June 2010 on the rocky shore of the Costa Brava close to the town of Blanes (41°67’N, 2°30’E) in the northwest Mediterranean Sea. 15 individuals (five territorial males, five sneaker males and five females) were collected for individual expression analysis. Eight additional individuals (three territorial males, three females and two sneaker males) were collected and used as a pooled sample for the *de novo* transcriptome assembly. All specimens were caught on the same day with small nets and put into large containers for transport back to the laboratory under the same conditions. Territorial males were collected from their nests and females and sneaker males were collected in the surrounding area. Total body length and male gonad weight was measured. The territorial males were 6.52 cm (+/- 0.44 cm) long, whereas the sneaker males were 6.06 cm (+/-0.27 cm) long and the territorial males’ gonads weighed 0.037 g (+/-0.007 g) and the sneaker males’ gonads weighed 0.066 g (+/-0.015 g). Due to the small variation observed we can assume that individuals of each phenotype were in the same reproductive stage and can be considered biological replicates. About an hour elapsed between collecting and processing. Fish were euthanized immediately arriving to the laboratory, all individuals still showing the same phenotypic coloration, snap frozen in liquid nitrogen, and stored at ─80°C. The sex was double-checked especially for sneaker males and females, as they are phenotypically similar, by verifying the presence of ovaries or testes. The collection of fish samples was conducted in strict accordance with Spanish and European regulations. The method of euthanasia followed the Spanish Laws (Royal Executive Order, 53/2013) for Animal Experimentation and was done at the animal research facility of the Spanish National Research Council with the approval of the Directorate of Research of the Spanish Government. The study was found exempt from ethics approval by the ethics commission of the University of Barcelona since, according to article 3.1 of the European Union directive (2010/63/UE) from the 22/9/2010, no approval is needed for fish sacrification with the purpose of tissue or organ analyses. Furthermore, the study species *Tripterygion delaisi* is not listed in CITES.

### Total RNA extraction and cDNA library construction

Fish brains were dissected out of the frozen heads, weighed and placed in TRI Reagent (Molecular Research Center). All *Tripterygion delaisi* brains weighed between seven and twenty milligrams. Tissues were homogenized in a Retsch homogenizer (MM200) at 25Hz for two intervals of 30 seconds. Phase separation was done with 1/10 volume of BCP Phase Separation Reagent (1-Bromo-3-Chloropropane, Molecular Research Center) and centrifuged for 15 minutes at 12.000 g. The RNA was precipitated with 1/2 volume of isopropanol and centrifuged at 12.000 g for 8 minutes. The total RNA pellet was washed twice with 75% ethanol by vortexing and centrifuging for 5 minutes at 7.500 g to then be dissolved in 50 ul of TE Buffer (pH 8.0).

Poly-A mRNA was purified using Dynabeads (Invitrogen) coated in Oligo(dT)_25_ following the manufacturer’s protocol, but adding a second wash-step. Random Hexamer Primers (Invitrogen) were added and incubated at 65°C for 5 minutes. First-strand cDNA was synthesized with SuperScript reverse transcriptase (Invitrogen) by incubating at 25°C for 10 minutes, followed by 50 minutes at 42°C and 15 minutes at 70°C. Second strand cDNA was synthesized using RNaseH (New England Biolabs (NEB)) and DNA Polymerase I (NEB), incubated for 2.5 hours at 16°C. cDNA was purified with the QIAquick PCR purification kit (Qiagen) and fragmented with dsDNA fragmentase (NEB) for 28 minutes at 37°C and purified with the same kit. Fragments were prepared for Illumina sequencing with NEB kits following the manufacturer’s instructions for each reagent module. Firstly, ends were repaired with the End Repair module using *Escherichia coli* ligase and End Repair enzyme mix. A single A-base was then added using the dA-Tailing module with Klenow DNA polymerase. Custom paired-end adapters with 4 bp barcodes (Integrated DNA Technologies) were added with the Quick T4 DNA ligase kit (Qiagen) to be able to multiplex the samples in the same Illumina lane. After each step the DNA was purified either with the PCR purification kit or the MiniElute PCR purification kit (Qiagen). Subsequently, 200-300 bp fragments were cut from a 2% ultra-pure agarose gel (Invitrogen), run for 60 minutes at 110 V, and cleaned with the QIAquick gel extraction kit (Qiagen). Finally, cDNA fragments were enriched using Phusion polymerase (NEB) and paired end PCR Primers (Integrated DNA Technologies). Enrichment PCR was performed as follows: initial denaturation at 98°C for 30 seconds, 15 cycles at 98°C for 10 seconds, 65°C for 30 seconds and 72°C for 30 seconds; and a final extension for 5 minutes at 72°C. PCR products were purified with the PCR purification kit (Qiagen) and resuspended with 30 μl of EB solution. Concentration and purity was measured several times throughout the process with the Agilent 2100 Bioanalyzer (Agilent RNA 6000 pico & DNA 1000 Kit).

Sequencing was conducted on an Illumina Hiseq 2000 using the Illumina 1.5 baseline calling pipeline. A duplex-specific nuclease (DSN) normalized pooled library of 8 mixed samples (three territorial males, three females and two sneaker males) were sequenced at the length of 109 bp, single-end, at DNAVision S.A. (Belgium). At the FAS Center for systems biology at Harvard University (USA) three individuals (one territorial male, one sneaker male and one female) were sequenced at the length of 109 bp and twelve individuals (four territorial males, four sneaker males and four females) were sequenced at a length of 52 bp, all single end. Reads sequenced at the length of 109 bp were used to assemble the reference transcriptome. The reason for the production of a normalized and pooled sample of 8 individuals was to increase the probability of sequencing rare genes. Reads sequenced from individual not-pooled samples, both at 109 bp and at 52 bp, were used for expression analysis.

### Read processing, de novo transcriptome assembly and annotation

Reads were sorted and the four-mer barcodes were removed using custom Perl Scripts in the FASTX-Toolkit ( http://hannonlab.cshl.edu/fastx_toolkit/). Read quality was checked and visualized with FastQC (Andrews 2010) and low quality reads were eliminated or trimmed in CLC Genomics Workbench 4.7 so that all base reads were superior to the Phred quality score of 35. Reads with the length below 20 bp were removed.

The d*e novo* assembly was performed with Trinity [28], which allows for the detection of differentially spliced contig isoforms, using the default program settings. Contigs shorter than 200 bp were eliminated. Contamination was identified by blasting the contigs (BLASTn) against locally installed databases from November 2012 (NCBI UniVec database and NCBI fungal, bacterial and viral genomes). Contigs with successful hits (E-value cut-off of 1×10^-25^) were removed from the assembly before further analysis. The transcripts/contigs in the de-contaminated assembly were blasted against a locally installed UniProt protein database (release 2011_11) using BLASTx (version 2.2.22) with an E-value cut-off of 1x10^-10^, a word-size of 3 and the BLOSUM62 matrix. Transcripts were also compared to the annotated proteins available on the NCBI Unigene database of *Danio rerio*. The de novo assembly contigs were divided into two sets: ‘with BLAST’, which are the contigs that resulted in a successful BLAST hit and ‘without BLAST’, which are the contigs without homology in the protein databases. Both sets were then compared. Open reading frame presence and length was measured by using the getorf tool implemented in the program EMBOSS 6.6.0.0 [62]. To detect the protein coding potential of the contigs we used the program CPAT v 1.2 [33], which is a coding potential assessment tool, and selected the Zebrafish (Zv9/danRer7) as the assembly database. We accepted contigs with a potential above 0.5 to be ‘more likely’ protein coding. Furthermore GC content and expression levels were compared between the two sets.

### RNAseq analysis and differential expression

All transcriptome contigs (‘with BLAST’ and ‘without BLAST’) were used as the reference trancriptome for the evaluation of the expression values. The three individual samples of 109 bp length (one territorial male, one sneaker male and one female) were cut down to 48 bp to avoid a length bias in the expression value calculation, as the other 12 individual samples (four territorial males, four sneaker males and four females) were sequenced at a shorter length. On average 13 million quality trimmed reads for each of the 15 individuals (Additional file 1: Table S1) were mapped against the reference transcriptome with Bowtie, a short read aligner (specific parameters: -n 2, -l 25, -m 200 and-a) [63]. RSEM v1.1.19 [64] was then used to quantify mapped reads by using the standard settings. Unmapped reads were discarded. Differential expression values were computed with EBseq [61] by using a Bayesian approach to accurately estimate isoform expression. Three comparisons were performed to find the particular genes which distinguish each phenotype: territorial males versus sneaker males, territorial males versus females and sneaker males versus females. Count data were normalized by estimating a scaling factor for each contig in EBseq, which has been demonstrated to be a very robust method [65]. We tested for differences between the normalized means with an empirical Bayes hierarchical model resulting in posterior probabilities of differential expression. Five iterations were run for each comparison. Comparisons were accepted to be significant at an FDR adjusted value of 0.05. To avoid outlier expression bias due to great individual variation, we accepted only contigs for which standard deviation was smaller than the mean expression value within phenotype (SD < Mean). For visualization of the significant comparisons, heatmaps of the significant genes after FDR adjustment were produced with the heatmaps2 package in R. Hierarchical clustering of individual samples with 1000 bootstrap replications was performed with the R package pvclust [66] and heatmaps were sorted accordingly. To visualize clusters of gene expression, we grouped the z-transformed expression ratios by using k-means in R.

### Functional annotation

The *de novo* assembled transcriptome was annotated with Blast2GO [35]. We performed several enrichment analyses. Firstly, the top 1% contigs which were expressed at the highest level regardless of phenotype were compared in an enrichment analyses against the whole transcriptome by the means of a Fisher’s Exact test. Enriched GO-terms were then slimmed in REVIGO and treemaps produced [67]. Secondly, enrichment analyses were performed for the differentially expressed genes for the three comparisons.

Genes previously reported to be of importance between phenotypes with alternative reproductive strategies in fish are here termed ‘candidate genes’ in the context of social dominance. The sequences of the candidate genes *gnrh* (gonadotropin releasing hormone receptor; [14,19]), *epd* (ependymin; [68]), *avp* (arginine vasopressin; [22,48,55,69]), *somatostatin*[14], *egr*1 (early growth response protein, [19]), *galn* (galanin, [14]) and *cyp19a1* (brain aromatase enzyme, [14]) were blasted (BLASTn) against the *de novo* assembly contigs using BLAST 2 sequence in NCBI to confirm their presence in the reference transcriptome and evaluate its differential expression among phenotypes.

### Availability of supporting data

This Transcriptome Shotgun Assembly project has been deposited at DDBJ/EMBL/GenBank under the accession GAJK00000000. The version described in this paper is the first version, GAJK01000000. Raw sequence reads can be found in the SRA database under BioProject PRJNA186408.

## Competing interests

The authors declare that they have no competing interests.

## Authors’ contributions

CS, SV, EM & MP conceived the idea and designed the research. CS & EM conducted the field work. CS conducted the lab work with help of SV. CS & MP analyzed the results and CS wrote the paper with support and comments by all the authors. All authors read and approved the final manuscripts.

## Supplementary Material

Additional file 1: Table S1-Table S7 **Table S1:** Size and number of reads (after quality trimming) for each sample. TM = territorial male, SM = sneaker male, F = female. **Table S2:** Enriched functions throughout the whole transcriptome of *Tripterygion delaisi* with elevated GO-term function and the clustered lower-level GO-terms (if applicable). The letter correspond to letters found in the treemap (Figure 2). **Table S3:** Genes expressed on average above 10000 reads throughout the transcriptome. Normalized expression values for all 15 individuals, as well as average and sums are provide **Table S4:** List of differentially expressed contigs with successful gene annotation between territorial male (TM) and sneaker male (SM) of *T. delaisi*. **Table S5:** List of differentially expressed contigs with successful gene annotation between sneaker male (SM) and female (F) of *T. delaisi*. **Table S6:** List of differentially expressed contigs with successful gene annotation between territorial male (TM) and female (F) of *T. delaisi*. **Table S7:** Posterior probability of differential expression of each ‘candidate gene’ isoform for each of the comparisons. Gene name and description as well as *T. delaisi* transcriptome contig identifier are provided. Probabilities marked in red were found to be significantly differentially expressed, but only the grey cell were differentially expressed after exclusion of differentially expressed contigs due to high individual variation (SD > Mean). Click here for file

Additional file 2: Figure S1 Read coverage for the de novo transcriptome assembly contigs (above) and weighted frequency distribution of coverage by the length of the transcriptome contigs (below). Click here for file

Additional file 3: Figure S2 Frequency distribution of isoforms detected in the de novo assembly of the reference transcriptome. (a) Amount of contigs with different number of isoforms (b) length of contigs with different number of isoforms. Click here for file

Additional file 4: Figure S3 Heatmap comparing significant differentially expressed contigs between five females and five territorial males. Intensity of color indicates expression levels. Similarity in expression patterns between genes is represented by kmeans clustering separating highly expressed genes above the white line and less expressed genes below. Similarity between individuals with hierarchical clustering can be seen above the heatmap with bootstraps. Click here for file

Additional file 5: Figure S4 Heatmap comparing significant differentially expressed contigs between five females and five sneaker males. Intensity of color indicates expression levels. Similarity in expression patterns between genes is represented by kmeans clustering separating highly expressed genes above the white line and less expressed genes below. Similarity between individuals with hierarchical clustering can be seen above the heatmap with bootstraps. Click here for file

## References

[B1] Gonçalves D Teles M Alpedrinha J Oliveira RF Brain and gonadal aromatase activity and steroid hormone levels in female and polymorphic males of the peacock blenny *Salaria pavo* Horm Behav 2008 54 717 25 10.1016/j.yhbeh.2008.07.014 18760279

[B2] Alonzo SH Warner RR A trade-off generated by sexual conflict: Mediterranean wrasse males refuse present mates to increase future success Behav Ecol 1999 10 105 111 10.1093/beheco/10.1.105

[B3] Munehara H Takenaka O Microsatellite markers and multiple paternity in a paternal care fish, Hexagrammos otakii J Ethol 2000 18 101 104 10.1007/s101640070007

[B4] Taborsky M Oliveira RF, Taborsky MBH Alternative reproductive tactics in fish Altern Reprod tactics an Integr approach 2008 Cambridge: Cambridge University Press 251 299

[B5] Taborsky M Sneakers, satellites, and helpers: parasitic and cooperative behavior in fish reproduction Adv Study Behav 1994 23 1 100

[B6] Taborsky M Sperm competition in fish: `bourgeois’ males and parasitic spawning Trends Ecol Evol 1998 13 222 227 10.1016/S0169-5347(97)01318-9 21238275

[B7] Immler S Mazzoldi C Rasotto MB From sneaker to parental male: change of reproductive traits in the black goby, *Gobius niger* (Teleostei, Gobiidae) J Exp Zool A Comp Exp Biol 2004 301 177 85 14743517 10.1002/jez.a.20019

[B8] Wirtz P The behaviour of the mediterranean tripterygion species (pisces, blennioidei) Z Tierpsychol 1978 48 142 174

[B9] De Jonge J Videler JJ Differences between the reproductive biologies of *Tripterygion tripteronotus* and *T. delaisi* (Pisces, Perciformes, Tripterygiidae): the adaptive significance of an alternative mating strategy and a red instead of a yellow nuptial colour Mar Biol 1989 100 431 437 10.1007/BF00394818

[B10] Pigliucci M Evolution of phenotypic plasticity: where are we going now? Trends Ecol Evol 2005 20 481 6 10.1016/j.tree.2005.06.001 16701424

[B11] Lande R Adaptation to an extraordinary environment by evolution of phenotypic plasticity and genetic assimilation J Evol Biol 2009 22 1435 46 10.1111/j.1420-9101.2009.01754.x 19467134

[B12] Thompson JD Phenotypic plasticity as a component of evolutionary change Trends Ecol Evol 1991 6 246 9 10.1016/0169-5347(91)90070-E 21232470

[B13] Thomas ML Simmons LW Short-term phenotypic plasticity in long-chain cuticular hydrocarbons Proc Biol Sci 2011 278 3123 8 10.1098/rspb.2011.0159 21367785 PMC3158943

[B14] Renn SCP Aubin-Horth N Hofmann HA Fish and chips: functional genomics of social plasticity in an African cichlid fish J Exp Biol 2008 211 Pt 18 3041 56 18775941 10.1242/jeb.018242 PMC3728697

[B15] Gabriel W How stress selects for reversible phenotypic plasticity J Evol Biol 2005 18 873 83 10.1111/j.1420-9101.2005.00959.x 16033559

[B16] Maruska KP Fernald RD Behavioral and physiological plasticity: rapid changes during social ascent in an African cichlid fish Horm Behav 2010 58 230 40 10.1016/j.yhbeh.2010.03.011 20303357 PMC2922674

[B17] Robinson GE Fernald RD Clayton DF Genes and social behavior Science 2008 322 896 900 10.1126/science.1159277 18988841 PMC3052688

[B18] Whitfield CW Cziko A-M Robinson GE Gene expression profiles in the brain predict behavior in individual honey bees Science 2003 302 296 9 10.1126/science.1086807 14551438

[B19] Burmeister SS Jarvis ED Fernald RD Rapid behavioral and genomic responses to social opportunity PLoS Biol 2005 3 e363 10.1371/journal.pbio.0030363 16216088 PMC1255743

[B20] Fernald RD Social control of the brain Annu Rev Neurosci 2012 35 133 51 10.1146/annurev-neuro-062111-150520 22524786

[B21] Aubin-Horth N Landry CR Letcher BH Hofmann HA Alternative life histories shape brain gene expression profiles in males of the same population Proc Biol Sci 2005 272 1655 62 10.1098/rspb.2005.3125 16087419 PMC1559854

[B22] Guiry A Flynn D Hubert S O’Keeffe AM LeProvost O White SL Forde PF Davoren P Houeix B Smith TJ Cotter D Wilkins NP Cairns MT Testes and brain gene expression in precocious male and adult maturing Atlantic salmon (*Salmo salar*) BMC Genomics 2010 11 211 10.1186/1471-2164-11-211 20350334 PMC2996963

[B23] Carreras-Carbonell J Macpherson E Pascual M Population structure within and between subspecies of the Mediterranean triplefin fish *Tripterygion delaisi* revealed by highly polymorphic microsatellite loci Mol Ecol 2006 15 3527 39 10.1111/j.1365-294X.2006.03003.x 17032255

[B24] Carreras-Carbonell J Macpherson E Pascual M Rapid radiation and cryptic speciation in mediterranean triplefin blennies (Pisces: Tripterygiidae) combining multiple genes Mol Phylogenet Evol 2005 37 751 61 10.1016/j.ympev.2005.04.021 15964768

[B25] Wang Z Gerstein M Snyder M RNA-Seq: a revolutionary tool for transcriptomics Nat Rev Genet 2009 10 57 63 10.1038/nrg2484 19015660 PMC2949280

[B26] Ekblom R Galindo J Applications of next generation sequencing in molecular ecology of non-model organisms Heredity (Edinb) 2011 107 1 15 10.1038/hdy.2010.152 21139633 PMC3186121

[B27] Lin H-C Hastings PA Phylogeny and biogeography of a shallow water fish clade (Teleostei: Blenniiformes) BMC Evol Biol 2013 13 210 10.1186/1471-2148-13-210 24067147 PMC3849733

[B28] Grabherr MG Haas BJ Yassour M Levin JZ Thompson DA Amit I Adiconis X Fan L Raychowdhury R Zeng Q Chen Z Mauceli E Hacohen N Gnirke A Rhind N di Palma F Birren BW Nusbaum C Lindblad-Toh K Friedman N Regev A Full-length transcriptome assembly from RNA-Seq data without a reference genome Nat Biotechnol 2011 29 644 52 10.1038/nbt.1883 21572440 PMC3571712

[B29] Barshis DJ Ladner JT Oliver TA Seneca FO Traylor-Knowles N Palumbi SR Genomic basis for coral resilience to climate change Proc Natl Acad Sci U S A 2013 110 1387 92 10.1073/pnas.1210224110 23297204 PMC3557039

[B30] Wang ET Sandberg R Luo S Khrebtukova I Zhang L Mayr C Kingsmore SF Schroth GP Burge CB Alternative isoform regulation in human tissue transcriptomes Nature 2008 456 470 6 10.1038/nature07509 18978772 PMC2593745

[B31] Stamm S Ben-Ari S Rafalska I Tang Y Zhang Z Toiber D Thanaraj TA Soreq H Function of alternative splicing Gene 2005 344 1 20 15656968 10.1016/j.gene.2004.10.022

[B32] Modrek B Lee C A genomic view of alternative splicing Nat Genet 2002 30 13 9 10.1038/ng0102-13 11753382

[B33] Wang L Park HJ Dasari S Wang S Kocher J-P Li W CPAT: Coding-Potential Assessment Tool using an alignment-free logistic regression model Nucleic Acids Res 2013 41 6 e74 10.1093/nar/gkt006 23335781 PMC3616698

[B34] Ferreira PG Patalano S Chauhan R Ffrench-Constant R Gabaldon T Guigo R Sumner S Transcriptome analyses of primitively eusocial wasps reveal novel insights into the evolution of sociality and the origin of alternative phenotypes Genome Biol 2013 14 R20 10.1186/gb-2013-14-2-r20 23442883 PMC4053794

[B35] Conesa A Götz S García-Gómez JM Terol J Talón M Robles M Blast2GO: a universal tool for annotation, visualization and analysis in functional genomics research Bioinformatics 2005 21 3674 6 10.1093/bioinformatics/bti610 16081474

[B36] Suárez-Castillo EC García-Arrarás JE Molecular evolution of the ependymin protein family: a necessary update BMC Evol Biol 2007 7 23 10.1186/1471-2148-7-23 17302986 PMC1805737

[B37] Thomson JS Watts PC Pottinger TG Sneddon LU Plasticity of boldness in rainbow trout, *Oncorhynchus mykiss*: do hunger and predation influence risk-taking behaviour? Horm Behav 2012 61 750 757 10.1016/j.yhbeh.2012.03.014 22498695

[B38] Oliveira RF Social plasticity in fish: integrating mechanisms and function J Fish Biol 2012 81 2127 2150 10.1111/j.1095-8649.2012.03477.x 23252731

[B39] Bent AF Plant mitogen-activated protein kinase cascades: Negative regulatory roles turn out positive Proc Natl Acad Sci U S A 2001 98 784 6 10.1073/pnas.98.3.784 11158543 PMC33365

[B40] Aubin-Horth N Renn SCP Genomic reaction norms: using integrative biology to understand molecular mechanisms of phenotypic plasticity Mol Ecol 2009 18 3763 80 10.1111/j.1365-294X.2009.04313.x 19732339

[B41] Thomas GM Huganir RL MAPK cascade signalling and synaptic plasticity Nat Rev Neurosci 2004 5 173 83 10.1038/nrn1346 14976517

[B42] Trainor BC Hofmann HA Somatostatin and somatostatin receptor gene expression in dominant and subordinate males of an African cichlid fish Behav Brain Res 2007 179 314 20 10.1016/j.bbr.2007.02.014 17374406 PMC2696992

[B43] Trainor BC Hofmann HA Somatostatin regulates aggressive behavior in an African cichlid fish Endocrinology 2006 147 5119 25 10.1210/en.2006-0511 16887916

[B44] Hofmann HA Fernald RD Social status controls somatostatin neuron size and growth J Neurosci 2000 20 4740 4 10844043 10.1523/JNEUROSCI.20-12-04740.2000 PMC6772449

[B45] Mayer I Borg B Berglund I Lambert JGD Effects of castration and androgen treatment on aromatase activity in the brain of mature male Atlantic salmon (*Salmo salar* L.) parr Gen Comp Endocrinol 1991 82 86 92 10.1016/0016-6480(91)90299-L 1874393

[B46] Nunez BS Applebaum SL Tissue- and sex-specific regulation of CYP19A1 expression in the Atlantic croaker (*Micropogonias undulatus*) Gen Comp Endocrinol 2006 149 205 16 10.1016/j.ygcen.2006.06.005 16872606

[B47] Grober MS George AA Watkins KK Carneiro LA Oliveira RF Forebrain AVT and courtship in a fish with male alternative reproductive tactics Brain Res Bull 2002 57 423 5 10.1016/S0361-9230(01)00704-3 11923002

[B48] Greenwood AK Wark AR Fernald RD Hofmann HA Expression of arginine vasotocin in distinct preoptic regions is associated with dominant and subordinate behaviour in an African cichlid fish Proc Biol Sci 2008 275 2393 402 10.1098/rspb.2008.0622 18628117 PMC2603226

[B49] Sanogo YO Band M Blatti C Sinha S Bell AM Transcriptional regulation of brain gene expression in response to a territorial intrusion Proc Biol Sci 2012 279 4929 38 10.1098/rspb.2012.2087 23097509 PMC3497248

[B50] Santangelo N Bass AH New insights into neuropeptide modulation of aggression: field studies of arginine vasotocin in a territorial tropical damselfish Proc Biol Sci 2006 273 3085 92 10.1098/rspb.2006.3683 17015351 PMC1679891

[B51] Kerr SC Azzouz N Fuchs SM Collart MA Strahl BD Corbett AH Laribee RN The Ccr4-Not complex interacts with the mRNA export machinery PLoS One 2011 6 e18302 10.1371/journal.pone.0018302 21464899 PMC3065485

[B52] Karsenty G Wagner EF Reaching a genetic and molecular understanding of skeletal development Dev Cell 2002 2 389 406 10.1016/S1534-5807(02)00157-0 11970890

[B53] Siderovski DP Willard FS The GAPs, GEFs, and GDIs of heterotrimeric G-protein alpha subunits Int J Biol Sci 2005 1 51 66 15951850 10.7150/ijbs.1.51 PMC1142213

[B54] Kimple AJ Soundararajan M Hutsell SQ Roos AK Urban DJ Setola V Temple BRS Roth BL Knapp S Willard FS Siderovski DP Structural determinants of G-protein alpha subunit selectivity by regulator of G-protein signaling 2 (RGS2) J Biol Chem 2009 284 19402 11 10.1074/jbc.M109.024711 19478087 PMC2740565

[B55] Larson ET O’Malley DM Melloni RH Aggression and vasotocin are associated with dominant-subordinate relationships in zebrafish Behav Brain Res 2006 167 94 102 10.1016/j.bbr.2005.08.020 16213035

[B56] Paterlini M Revilla V Grant AL Wisden W Expression of the neuronal calcium sensor protein family in the rat brain Neuroscience 2000 99 205 16 10.1016/S0306-4522(00)00201-3 10938426

[B57] Kikkawa U Matsuzaki H Yamamoto T Protein Kinase C (PKC): Activation Mechanisms and Functions J Biochem 2002 132 831 839 10.1093/oxfordjournals.jbchem.a003294 12473183

[B58] Berger A Roberts MA Berger A, Roberts MA Dietary Effects of Arachidonate-Rich Fungal Oil and Fish Oil on Murine Hippocampal Gene Expression Unraveling Lipid Metab with Microarrays 2005 New York: Marcel Dekker 69 94 10.1186/1476-511X-1-2 PMC139963 12617750

[B59] Gonzàlez-Porta M Calvo M Sammeth M Guigó R Estimation of alternative splicing variability in human populations Genome Res 2012 22 528 38 10.1101/gr.121947.111 22113879 PMC3290788

[B60] Mochizuki N Ohba Y Kiyokawa E Kurata T Murakami T Ozaki T Kitabatake A Nagashima K Matsuda M Activation of the ERK/MAPK pathway by an isoform of rap1GAP associated with G alpha(i) Nature 1999 400 891 4 10.1038/23738 10476970

[B61] Leng N Dawson JA Thomson JA Ruotti V Rissman AI Smits BMG Haag JD Gould MN Stewart RM Kendziorski C EBSeq: An empirical Bayes hierarchical model for inference in RNA-seq experiments Bioinformatics 2013 1035 1043 23428641 10.1093/bioinformatics/btt087 PMC3624807

[B62] Rice P Longden I Bleasby A EMBOSS: the european molecular biology open software suite Trends Genet 2000 16 276 7 10.1016/S0168-9525(00)02024-2 10827456

[B63] Langmead B Trapnell C Pop M Salzberg SL Ultrafast and memory-efficient alignment of short DNA sequences to the human genome Genome Biol 2009 10 R25 10.1186/gb-2009-10-3-r25 19261174 PMC2690996

[B64] Li B Ruotti V Stewart RM Thomson JA Dewey CN RNA-Seq gene expression estimation with read mapping uncertainty Bioinformatics 2010 26 493 500 10.1093/bioinformatics/btp692 20022975 PMC2820677

[B65] Dillies M-A Rau A Aubert J Hennequet-Antier C Jeanmougin M Servant N Keime C Marot G Castel D Estelle J Guernec G Jagla B Jouneau L Laloë D Le Gall C Schaëffer B Le Crom S Guedj M Jaffrézic F A comprehensive evaluation of normalization methods for Illumina high-throughput RNA sequencing data analysis Brief Bioinform 2012 1 13 10.1093/bib/bbs046 22988256

[B66] Suzuki R Shimodaira H Pvclust: an R package for assessing the uncertainty in hierarchical clustering Bioinformatics 2006 22 1540 2 10.1093/bioinformatics/btl117 16595560

[B67] Supek F Bošnjak M Škunca N Šmuc T REVIGO summarizes and visualizes long lists of gene ontology terms PLoS One 2011 6 e21800 10.1371/journal.pone.0021800 21789182 PMC3138752

[B68] Sneddon LU Schmidt R Fang Y Cossins AR Molecular correlates of social dominance: a novel role for ependymin in aggression PLoS One 2011 6 e18181 10.1371/journal.pone.0018181 21483679 PMC3071721

[B69] Miranda JA Oliveira RF Carneiro LA Santos RS Grober MS Neurochemical correlates of male polymorphism and alternative reproductive tactics in the Azorean rock-pool blenny, *Parablennius parvicornis* Gen Comp Endocrinol 2003 132 183 189 10.1016/S0016-6480(03)00063-7 12812764

