# Supplementary figures and images for: Transcriptome analyses and differential gene expression in a non-model fish species with alternative mating tactics
Source: BMC Genomics. 2014 Feb 28;15:167. doi: 10.1186/1471-2164-15-167 (PMC4029132; doi:10.1186/1471-2164-15-167)

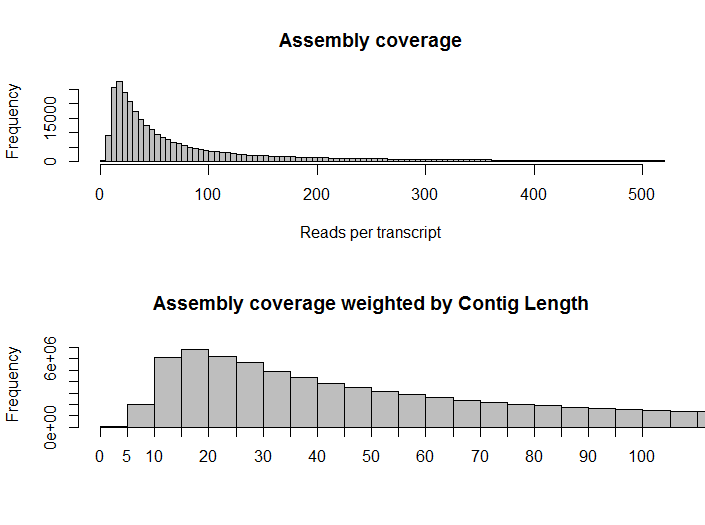

Supplement: Additional file 2: Figure S1 — Read coverage for the de novo transcriptome assembly contigs (above) and weighted frequency distribution of coverage by the length of the transcriptome contigs (below). [file 1471-2164-15-167-S2.PNG]

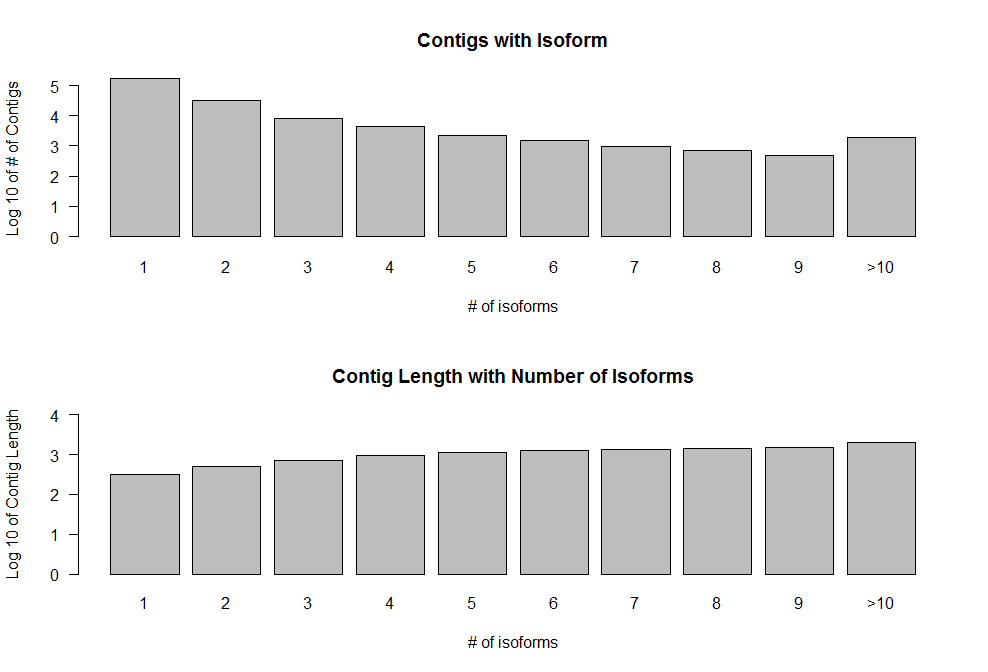

Supplement: Additional file 3: Figure S2 — Frequency distribution of isoforms detected in the de novo assembly of the reference transcriptome. (a) Amount of contigs with different number of isoforms (b) length of contigs with different number of isoforms. [file 1471-2164-15-167-S3.PNG]

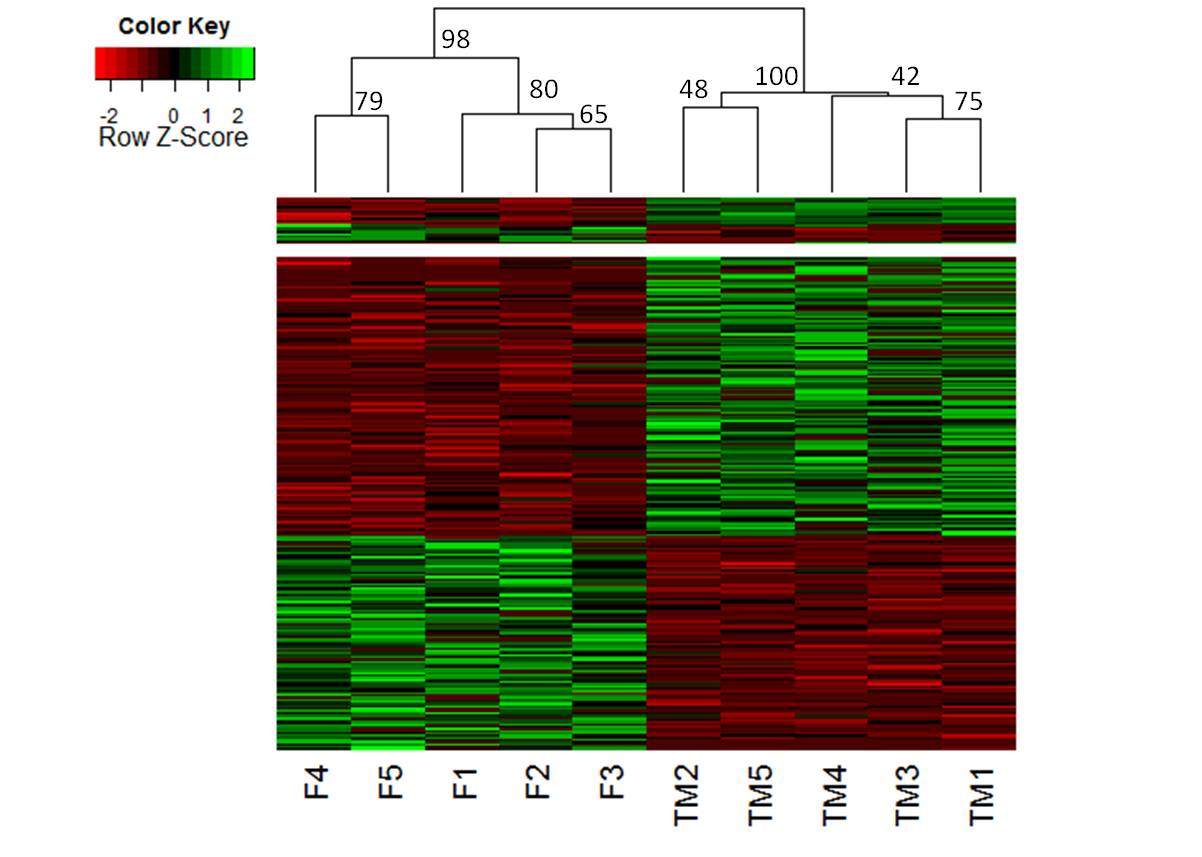

Supplement: Additional file 4: Figure S3 — Heatmap comparing significant differentially expressed contigs between five females and five territorial males. Intensity of color indicates expression levels. Similarity in expression patterns between genes is represented by kmeans clustering separating highly expressed genes above the white line and less expressed genes below. Similarity between individuals with hierarchical clustering can be seen above the heatmap with bootstraps. [file 1471-2164-15-167-S4.PNG]

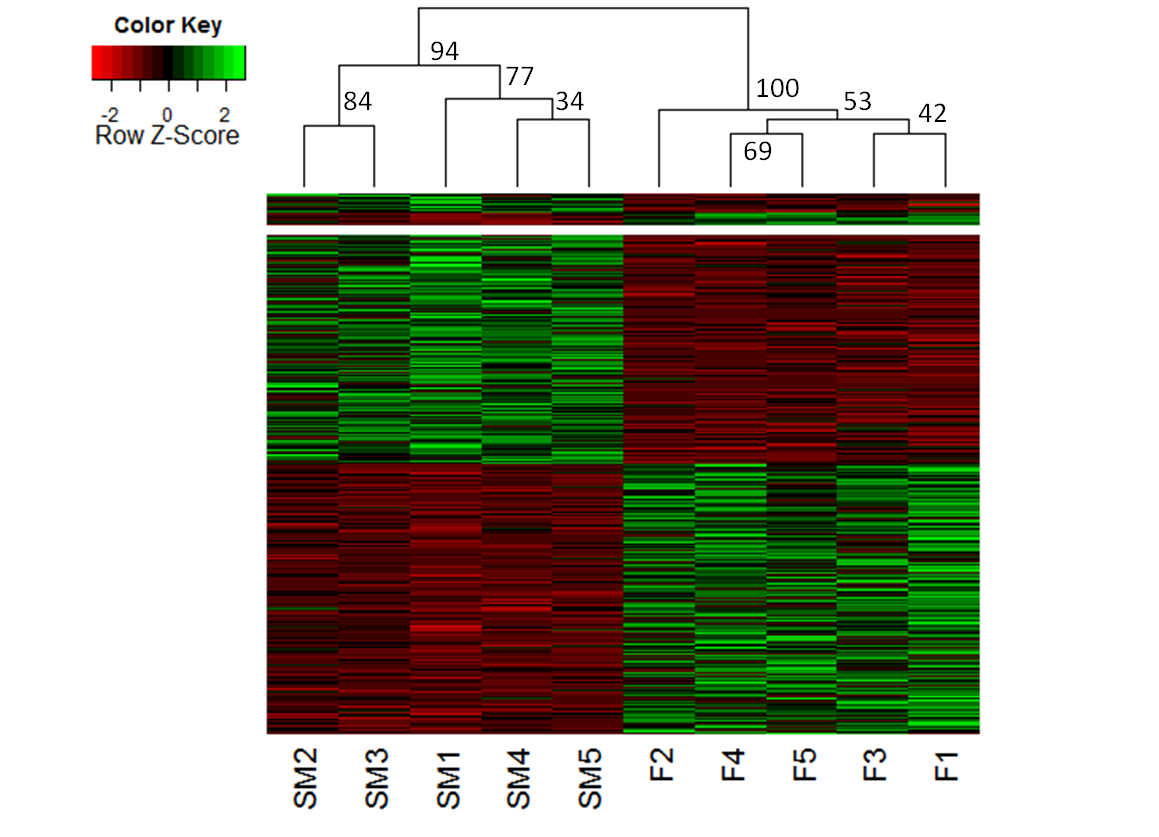

Supplement: Additional file 5: Figure S4 — Heatmap comparing significant differentially expressed contigs between five females and five sneaker males. Intensity of color indicates expression levels. Similarity in expression patterns between genes is represented by kmeans clustering separating highly expressed genes above the white line and less expressed genes below. Similarity between individuals with hierarchical clustering can be seen above the heatmap with bootstraps. [file 1471-2164-15-167-S5.PNG]
